# Adductor Canal Block Versus Local Infiltration Analgesia for Postoperative Pain Management in Total Knee Arthroplasty

**DOI:** 10.7759/cureus.57408

**Published:** 2024-04-01

**Authors:** Mohd J Khan, Nazia Tauheed, Anwar H Siddiqui, Amir B Sabir, Shahla Haleem

**Affiliations:** 1 Department of Anaesthesiology and Critical Care, Jawaharlal Nehru Medical College and Hospital, Aligarh Muslim University, Aligarh, IND; 2 Department of Physiology, Jawaharlal Nehru Medical College and Hospital, Aligarh Muslim University, Aligarh, IND; 3 Department of Orthopaedic Surgery, Jawaharlal Nehru Medical College and Hospital, Aligarh Muslim University, Aligarh, IND; 4 Department of Anaesthesiology, Jawaharlal Nehru Medical College and Hospital, Aligarh Muslim University, Aligarh, IND

**Keywords:** total knee arthroplasty (tka), postoperative pain, periarticular injection, local infiltration analgesia (lia), adductor canal block (acb)

## Abstract

Background: Total knee arthroplasty (TKA) is a common surgical procedure for patients with knee osteoarthritis, often associated with postoperative pain. Effective pain management strategies are essential for improving patient outcomes and satisfaction. This study aimed to compare the efficacy of two analgesic modalities, local infiltration analgesia (LIA) and adductor canal block (ACB), in providing postoperative pain relief for patients undergoing TKA.

Methods: This prospective randomized comparative study included 60 patients undergoing TKA for knee osteoarthritis under subarachnoid block (spinal anaesthesia). Patients were divided into two groups: LIA group (local wound infiltration with periarticular injection of bupivacaine 0.125% + dexmedetomidine 1 mcg/kg) and ACB group (ACB with bupivacaine 0.125% + 1 mcg/kg dexmedetomidine). Pain relief was assessed using the Numerical Rating Scale (NRS) score, time to first rescue analgesic requirement (NRS > 3), and total amount of analgesic needed in the first 24 hours post-surgery.

Results: The time to first perception of pain with NRS > 3 was 11.30±0.8 hours in the ACB group and 9.40 ± 1.1 hours in the LIA group, with a statistically significant difference (p < 0.001). Additionally, the total number of rescue analgesic doses given in the first 24 hours post-operatively differed significantly between the two groups (p = 0.046).

Conclusion: The study concludes that ACB is an effective postoperative analgesic modality, superior to local infiltration analgesia, for patients undergoing TKA.

## Introduction

Osteoarthritis (OA) stands as the predominant form of joint arthritic disease, prominently affecting weight-bearing joints including the knee, hip, cervical, and lumbosacral spine. This condition manifests through progressive breakdown of joint cartilage and bone, leading to symptoms like joint pain, swelling, stiffness, and eventual deformity. Globally, knee OA affects approximately 16% of individuals aged 15 and above, rising to 22.9% in those aged 40 years and older [1]. In India, about 80% of individuals reporting knee pain are diagnosed with OA, with 20% experiencing limitations in daily activities and 11% requiring specialized care [2,3].

Management strategies for OA focus on patient education, pain management, functional enhancement, and disease process modulation. Current therapeutic modalities encompass patient education, exercise regimens, weight management, physiotherapy, and pharmacotherapy [4]. Surgical interventions, such as total knee arthroplasty (TKA), are primarily reserved for advanced degenerative knee joint disease [5].

Despite its efficacy, TKA is associated with notable adverse effects, including moderate to severe postoperative immobility and delayed rehabilitation, which in turn increase the risk of deep vein thrombosis and pulmonary embolism. Furthermore, up to 20% of TKA patients report chronic pain post-surgery, with inadequate pain management during intraoperative and postoperative periods being a major risk factor [6].

To address postoperative pain following TKA, various interventions and management strategies have been employed, including pre-emptive analgesia, local infiltration analgesia (LIA), systemic analgesics, neuraxial analgesia, and regional nerve blocks [7]. While neuraxial techniques, particularly continuous epidural analgesia, were once popular, their usage is declining due to limitations such as unintended motor blockade, bowel and bladder dysfunction, and hemodynamic instability [8].

In light of these challenges, regional nerve blocks have emerged as a favoured technique for post-neuraxial anaesthesia, offering early functional recovery, effective pain control, and shorter hospitalization. Notably, adductor canal block (ACB) and saphenous nerve block have garnered attention for their efficacy in providing analgesia post-TKA, while minimizing adverse effects associated with other techniques [9].

ACB involves the injection of local anaesthetic into the adductor canal, providing analgesia for knee, ankle, and foot surgeries. ACB selectively blocks sensory nerves while sparing motor function, offering advantages over other nerve block techniques. Recent studies have shown ACB to facilitate faster mobilization and functional recovery compared to femoral nerve block (FNB), without compromising analgesia [10,11].

LIA has also gained traction as a post-TKA pain management strategy. LIA, comprising wound infiltration combined with intraarticular injection of multimodal drugs, offers simplicity, shorter procedural time, and improved mobilization compared to traditional nerve blocks [12].

To address the need for optimized postoperative pain management in TKA patients, this study aimed to compare the efficacy of LIA (bupivacaine 0.125% + dexmedetomidine 1 mcg/kg) with ACB (bupivacaine 0.125% + 1 mcg/kg dexmedetomidine) in a randomized controlled trial [13]. This combination has been chosen due to its potential advantages in enhancing the duration and efficacy of analgesia, as supported by the literature on regional and neuraxial blocks [14]. Dexmedetomidine, an alpha-2 adrenergic agonist, possesses analgesic properties and has been shown to prolong the duration of sensory blockade when used as an adjuvant to local anaesthetics. Bupivacaine, a long-acting local anaesthetic, provides the main analgesic effect. By combining these agents, we aimed to provide superior pain relief compared to conventional approaches, thereby improving patient outcomes and satisfaction. The primary objective was to assess pain relief using the Numerical Rating Scale (NRS) score, with secondary objectives including time to first rescue analgesic requirement, total rescue analgesic consumption in the first 24 hours, and overall patient satisfaction score. 

## Materials and methods

Study design

This prospective, randomized, single-blinded study, with blinding applied to the patients, received approval from the Institutional Ethics Committee and was registered with the Clinical Trials Registry India (registration number CTRI/2021/07/035157).

Participants

Sixty patients undergoing TKA for knee OA under subarachnoid block (spinal anaesthesia) were enrolled. Inclusion criteria comprised American Society of Anesthesiologists (ASA) class I and II patients of both sexes, aged 18-75 years, weighing between 55-100 kg. Exclusion criteria included patients with mental dysfunction or cognitive deficiency, history of psychiatric illness, neuromuscular, vestibular, hepatic, or major systemic diseases, alcohol or drug abuse history, allergy to local anaesthetic, medical conditions affecting balance and coordination, lack of consent, and neuropathies.

Sample size calculation

The sample size was determined based on a pilot study, conducted over a two-month period prior to the current study (ethics committee ref no: 79/FM/IEC) involving 10 patients, with a minimum difference in pain scores of 30%. A minimum of 28 patients were required to achieve α=0.05 and β=0.20 with 80% power. Accounting for a 5-10% nonadherence rate, 40 patients were included in each group, totalling 80 patients.

Randomization

Patients were randomly allocated into two groups, each comprising 40 patients, using a computer-generated randomization sequence. This ensured that each participant had an equal chance of being assigned to either the ACB group or the LIA group. The randomization sequence was generated by a statistician who was not involved in the patient recruitment process, thus maintaining allocation concealment and minimizing selection bias.

Interventions

Pre-anesthetic evaluation and informed consent were obtained from all patients. Anaesthesia was induced via subarachnoid block at the L2-L3 or L3-L4 intervertebral space using hyperbaric bupivacaine (0.5%) 15 mg.

In one study group, ultrasonography-guided ACB was performed post-surgery using bupivacaine 0.125% + dexmedetomidine 0.5 mcg/kg (total volume 20 ml). A high-frequency linear probe was placed at the mid-thigh level (halfway between the anterior superior iliac spine and the patella; the femoral artery was identified under the sartorius muscle and confirmed using colour Doppler mode. Stimuplex® A needle (21G, 100 mm length, B. Braun, Melsungen, Germany) was introduced in plane and advanced into the adductor canal, its correct placement confirmed by injecting 2-3ml saline + saline spreading in the vicinity of the nerve. After confirmation, 20 ml of the medication as described above was injected into the adductor canal. 

In the other group, wound infiltration and periarticular injection were administered by the operating surgeon using bupivacaine 0.125% + dexmedetomidine 0.5 mcg/kg (total volume 40 ml).

Postoperative analgesia consisted of standard institutional protocol medications.

Outcomes

NRS, a validated tool was used to measure pain intensity [15]. Patients are asked to rate their pain on a scale from 0 (no pain) to 10 (worst pain imaginable). Time to first perception of pain (NRS > 3) is an outcome that measures the duration after surgery until the patient experiences moderate pain (NRS exceeding 3). A longer time to first perception of moderate pain indicates better pain control. Patients were monitored for complications and assessed for time to first perception of pain (NRS > 3), NRS score at this time, and total rescue analgesic doses given in 24 hours. Patient satisfaction was measured by the Short Assessment of Patient Satisfaction (SAPS) score. SAPS is a validated questionnaire that measures a patient's overall satisfaction with their post-operative pain management experience. It typically includes questions about pain intensity, control, and a patient's overall satisfaction with the pain management strategy [16]. Standard perioperative monitoring was conducted, with specific attention to complications such as bradycardia, hypotension, nausea, and vomiting.

Statistical analysis

Data analysis was conducted using IBM SPSS Statistics for Windows, Version 25, (Released 2017; IBM Corp., Armonk, New York, United States). Continuous variables were presented as mean ± standard deviation (SD) or median (interquartile range, IQR) depending on the distribution, while categorical variables were presented as frequencies and percentages. The primary outcome measure, time to first perception of pain (NRS > 3) and the secondary outcome measures, including NRS score at the time of first perception of pain, total rescue analgesic doses given in 24 hours (NRS > 3), and patient satisfaction score (SAPS), were compared between the groups using appropriate parametric (unpaired 't' test) or non-parametric tests (Mann-Whitney U test) depending on the distribution of the data. The normalcy of the data was assessed by Kolmogorov-Smirnov tests. All statistical tests were two-tailed, and a p-value < 0.05 was considered statistically significant.

## Results

The demographic data and baseline parameters, including age, sex, weight, height, ASA grading, type of surgery (unilateral or bilateral), and comorbidities, were recorded. Intergroup differences obtained were found to be statistically insignificant (p > 0.05). Both study groups were compared in terms of their hemodynamic and respiratory parameters (baseline and post-procedure), with intergroup differences being insignificant (p > 0.05) (Table 1).

**Table 1:** The baseline demographic and vital parameters in the study groups. SpO2: Saturation of peripheral oxygen; PR: Pulse rate; ASA-PS: American Society of Anesthesiologists physical status; TKA: Total knee arthroplasty

	Group A: Adductor canal block (ACB) group	Group B: Local infiltration analgesia (LIA) group	p-value
Number	40	40	
Male: female	23:17	25:15	0.219
Age (years)	59.9 ± 4.8	60.3±5.8	0.738
Weight (kg)	63.7 ± 3.92	64.8 ± 4.3	0.349
Height (m)	1.56 ± 0.07	1.58 ± 0.08	0.307
Mean arterial pressure (mmHg)	94.67 ± 5.0	96.30 ± 3.8	0.523
PR beats/min	83.4 ± 10.9	84.6 ± 7.8	0.705
SpO2 (%)	99.1 ± 1.1	98.9 ± 1.3	0.510
ASA-PS I (n (%))	21	18	0.606
ASA-PS II (n (%))	19	22	0.606
Surgery performed (n (%))	Right TKA – 12 (30%)	Right TKA – 15 (37%)	
	Left TKA – 08 (20%)	Left TKA – 13 (33%)	
	Bilateral TKA – 20 (50%)	Bilateral TKA – 12 (30%)	

NRS scores

**Figure 1:**
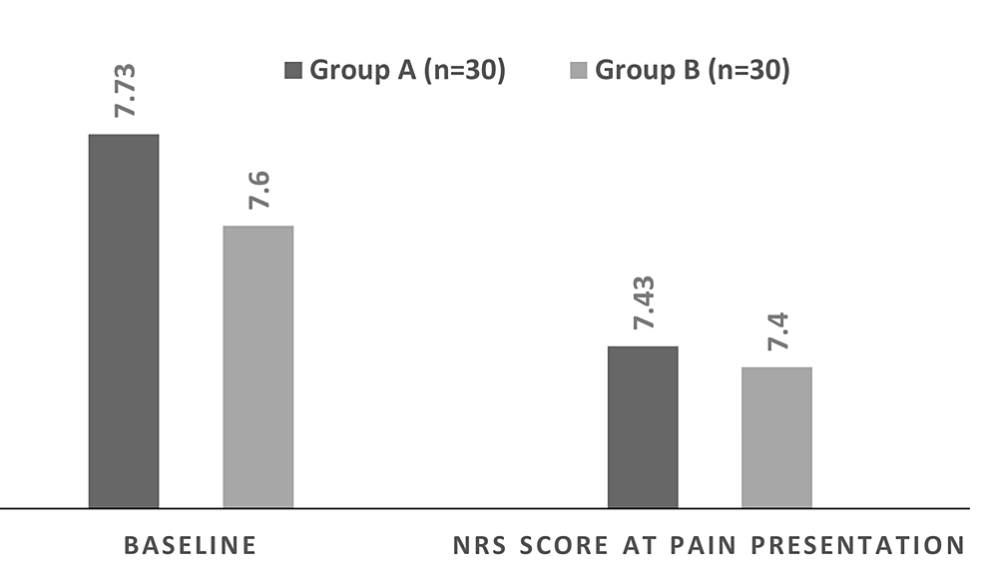
NRS scores at baseline and at pain presentation in both groups. Group A: Adductor canal block (ACB) group; Group B: Local infiltration analgesia (LIA) group NRS: Numerical Rating Scale

Pain scores at baseline and at the first presentation post-operatively were similar, with minimal intergroup differences that were statistically insignificant (p-values 0.497 and 0.881, respectively) (Figure 1).

Time to the first perception of pain (hours) / rescue analgesia in the first 24 hours postoperative

The time interval from the administration of the block to the first complaint of post-operative pain (NRS > 3) was calculated. Intergroup differences showed a shorter time to pain perception in group B, with this difference being statistically significant (p < 0.05). The total number of doses of rescue analgesic required in the first 24 hours post-operatively was less for group A patients. This intergroup difference was found to be statistically significant (p < 0.05). The mean sedation score was also comparable among the groups (p = 0.433) (Table 2).

**Table 2:** The time to first rescue analgesia (hours), the dosage of rescue analgesia, and the mean sedation score in both study groups postoperatively.

	Group A: adductor canal block (ACB) group	Group B: local infiltration analgesia (LIA) group	p-value
Time to first rescue analgesia (hours)	11.30 ± 0.8	9.40 ± 1.1	0.001
Rescue analgesic doses in the first 24 hours post-operative (number)	1.47 ± 0.4	1.77 ± 0.7	0.046
Mean sedation (Ramsay score)	3.60 ± 1.2	3.17 ± 0.9	0.433

Complications

Complications were minimal and comparable between the two groups. Nausea and sedation were common complications in both groups, but the difference was statistically insignificant (p > 0.05). The mean satisfaction score of patients at 24 hours post-surgery was compared. Although scores were slightly higher in group A compared to group B, this difference was found to be statistically insignificant (p > 0.05) (Table 3). 

**Table 3:** The mean satisfaction score and complications in both study groups postoperatively. LA: Local anaesthesia

		Group A: adductor canal block (ACB) group (n = 40)	Group B: local infiltration analgesia (LIA) group (n = 40)	p-value
Mean satisfaction score		21.6 ± 2.3	21.2 ± 2.6	0.578
Nausea and vomiting:	none	26	20	0.426
	nausea	12	16	
	vomiting	2	4	
Pruritis:	none	28	18	0.376
	mild	7	12	
	moderate	5	9	
	severe	0	1	
Hypotension		0	0	-
Bradycardia		0	0	-
LA toxicity		0	0	--
Distal neuro-vascular deficit		0	0	

## Discussion

This study compared the effectiveness of ultrasonography-guided ACB with bupivacaine and dexmedetomidine versus LIA for postoperative pain management after TKA. Key parameters such as NRS score post-operatively, time to first perception of pain/rescue analgesic, total number of rescue analgesic doses in the first 24 hours after surgery, satisfaction score, and associated complications were evaluated. The two groups were similar in baseline characteristics and hemodynamic parameters. Additionally, variations in hemodynamic parameters during and after the procedure were comparable between the groups.

While the initial pain scores were comparable between the groups, the time to first pain perception exceeding NRS 3 was significantly longer in the ACB group (11.30 ± 0.8 hours) compared to the LIA group (9.40 ± 1.1 hours) (p < 0.001). Consequently, patients receiving ACB required fewer rescue analgesic doses within the first 24 hours post-surgery (1.47 ± 0.4 vs. 1.77 ± 0.7; p = 0.046). These findings align with studies by Kampitak et al. [17] and Tong et al. [18], who observed reduced morphine consumption with ACB compared to LIA for postoperative pain after TKA. 

However, our results contrast with those of Marya et al. [19], who reported superior pain relief and lower fentanyl consumption with LIA at 12 and 24 hours postoperatively, and Grosso et al. [20], who found higher analgesic requirements in the ACB group compared to periarticular infiltration (PAI). These discrepancies might be due to variations in study design, sample size, and specific anaesthetic techniques.

Patient satisfaction scores were also measured and found to be similar between the two groups. A possible explanation could be that both interventions provided adequate pain relief within the first 24 hours post-surgery, which may have positively influenced patient satisfaction scores. Additionally, factors such as effective communication between healthcare providers and patients, preoperative education about pain management expectations, and overall quality of perioperative care could have contributed to similar satisfaction levels despite differences in pain control strategies. Additionally, complications were minimal and comparable between the groups, indicating the safety and feasibility of both approaches.

The potential benefits of ACB over LIA for post-operative pain management after TKA can be attributed to several anatomical and physiological factors. ACB specifically targets the saphenous nerve, which innervates the medial aspect of the knee joint and surrounding soft tissues. This targeted approach minimizes the spread of the local anaesthetic to other structures, potentially reducing the risk of motor block and other unintended side effects compared to LIA, which involves infiltrating a wider area [21]. ACB blocks sensory fibres responsible for pain perception, providing analgesia for the medial aspect of the knee, a key area involved in postoperative pain after TKA. By effectively blocking pain signals, ACB may lead to a decrease in reliance on opioid medications, which can have undesirable side effects like nausea, constipation, and respiratory depression [22].

This study employed dexmedetomidine as an adjuvant to bupivacaine in both ACB and LIA. While Goyal et al. [23] reported the benefits of adding dexmedetomidine to local anaesthetic in ACB compared to local anaesthetic alone, no studies comparing its use in LIA were found. The addition of dexmedetomidine to the LIA offers several potential advantages compared to using the standard LIA drug mixture. Firstly, dexmedetomidine has been shown to have a synergistic effect with local anaesthetics, resulting in prolonged analgesia and reduced opioid consumption postoperatively. This could lead to improved pain control and enhanced patient satisfaction following TKA surgery. Secondly, dexmedetomidine has sedative and anxiolytic properties, which may contribute to a smoother perioperative experience for patients by reducing anxiety and promoting relaxation. This could lead to improved patient comfort and potentially facilitate early ambulation and rehabilitation postoperatively. Studies investigating the use of dexmedetomidine as an adjuvant to local anaesthetics in various regional anaesthesia techniques have consistently demonstrated favourable outcomes, including prolonged duration of analgesia and reduced opioid requirements. Therefore, based on the known pharmacological properties of dexmedetomidine and the rationale for its use in LIA, we believe that its inclusion in the LIA mixture has the potential to improve postoperative pain management in TKA patients.

Limitation

This study has limitations, including the relatively small sample size and short follow-up duration (24 hours). A larger scale study with a longer follow-up time would be beneficial to confirm the findings and assess the impact on functional outcomes. It's important to note that ACB was administered after completion of the surgery, while LIA was administered by surgeons before closure. This difference in timing may have influenced the observed outcomes and should be considered when interpreting the results.

Future directions

Future research could explore the efficacy of ACB with dexmedetomidine compared to other pain management modalities for TKA, and investigate the long-term effects of this approach on pain management and functional recovery.

## Conclusions

Our findings suggest that ACB with bupivacaine and dexmedetomidine might offer advantages over LIA for postoperative pain management after TKA, providing a longer duration of analgesia and requiring fewer rescue analgesics within the first 24 hours. However, patient satisfaction and complication rates were similar between the groups. The choice between the two approaches may depend on factors such as patient preference, availability of resources, and clinician expertise. Further research with larger sample sizes and longer follow-ups is necessary to solidify these findings and establish the long-term benefits of this technique.
